# H3K9me3 and H4K20me3 represent the epigenetic landscape for 53BP1 binding to DNA lesions

**DOI:** 10.18632/aging.101572

**Published:** 2018-10-11

**Authors:** Alena Svobodová Kovaříková, Soňa Legartová, Jana Krejčí, Eva Bartová

**Affiliations:** 1 Institute of Biophysics of the Czech Academy of Sciences, Brno, 61265, Czech Republic

**Keywords:** Suv39h1/h2, H4K20me1/me2/me3, H3K9me3, DNA damage, epigenetics

## Abstract

Methylation of histones H4 at lysine 20 position (H4K20me), which is functional in DNA repair, represents a binding site for the 53BP1 protein. Here, we show a radiation-induced increase in the level of H4K20me3 while the levels of H4K20me1 and H4K20me2 remained intact. H4K20me3 was significantly pronounced at DNA lesions in only the G1 phase of the cycle, while this histone mark was reduced in very late S and G2 phases when PCNA was recruited to locally micro-irradiated chromatin. H4K20me3 was diminished in locally irradiated Suv39h1/h2 double knockout (dn) fibroblasts, and the same phenomenon was observed for H3K9me3 and its binding partner, the HP1β protein. Immunoprecipitation showed the existence of an interaction between H3K9me3-53BP1 and H4K20me3-53BP1; however, HP1β did not interact with 53BP1. Together, H3K9me3 and H4K20me3 represent epigenetic markers that are important for the function of the 53BP1 protein in non-homologous end joining (NHEJ) repair. The very late S phase represents the cell cycle breakpoint when a DDR function of the H4K20me3-53BP1 complex is abrogated due to recruitment of the PCNA protein and other DNA repair factors of homologous recombination to DNA lesions.

## Introduction

In recent years, it has been shown that histone modifications regulate gene expression and that these epigenetic processes are independent of changes in nucleotide sequences. Knowledge of epigenetic profiles is essential for an understanding of not only gene expression but also other cellular events, including regulation of the cell cycle, DNA repair, and apoptosis. Here, we studied the epigenetics of DNA repair and addressed the function of Suv39h1/h2 histone methyltransferases (HMTs) in the DNA damage response (DDR). In this regard, it is known that DNA lesions can occur spontaneously due to replication collisions or telomere dysfunction. In addition, DNA damage foci appear when the cells are exposed to radiation or DNA-damaging agents, and such DNA lesions can consist of double-strand breaks (DSBs) that are primarily deleterious to genome stability [1–3]. An injury in chromatin induces reorganization of the cell nucleus and causes local changes in the nuclear architecture. For example, chromosome territories (CTs) are rearranged and the local motion of promyelocytic leukemia (PML) bodies or Cajal bodies (CBs) is changed when the cells are exposed to radiation [4–7].

Considering DNA repair epigenetics, phosphorylation of histone H2AX, which is mediated by the ataxia-telangiectasia mutated (ATM) protein kinase, is a well-known marker of DSBs [8,9]. Similarly, di-methylation on histone H4 at the lysine 20 position (H4K20me2) and H3K79me2 contribute to the recruitment of the central DNA repair protein 53BP1 to chromatin in a vicinity of DSBs [10,11]. Thus, H4K20me2 seems to be an important histone marker in DNA repair processes, but on the other hand, H4K20me3 was found to be preferentially involved in the regulation of gene silencing when it appears at gene promoters or transposons [12–14]. From the view of DNA repair, it is well-known that H4K20me2 is recognized by a Tudor domain of the 53BP1 protein [11]. H4K20me2 is also considered to be an epigenetic hallmark required for the functional accumulation of the checkpoint protein Crb2 to sites of DNA damage [14]. Moreover, it has been reported that not only the histone methyltransferase Suv4-20h but also MMSET/WHSC1 is responsible for an increase of H4K20me2 and, thus, mediates the binding of the 53BP1 protein to H4K20 methylation [15,16]. Interestingly, recruitment of the 53BP1 protein to chromatin with DNA lesions can be affected by an inhibitor of Suv4-20h, A-196, which represents epi-drug blocking H4K20me2 and H4K20me3 but does not affect the phosphorylation of histone H2AX (γH2AX) [17]. This inhibitory effect reduced H4K20me2 and 53BP1 levels in parallel with an increase in H4K20me1 [18]. This observation supports the fact that H4K20me2 anchors the 53BP1 protein exactly at DNA lesions. Pellegrino et al. [19] additionally showed that the ability of 53BP1 to recognize chromatin with DSBs decreases in the S phase of the cell cycle, which is caused by the replication-coupled dilution of H4K20me2. Moreover, Simonetta et al. [20] showed that MAD2L2 (a homolog of yeast mitotic arrest-deficient protein, Mad2) accumulates at DSBs in DNA, which are associated with H4K20 di-methylated histones. This DDR-related nuclear event leads to the formation of the protein complex consisting of MAD2L2, 53BP1 and RAP1-interacting factor (RIF1). This protein complex suppresses the function of the BRCA1 protein in DNA lesions and, thus, potentiates the NHEJ repair mechanism. Drané et al. [21] showed that DNA damage stimulates phosphorylation of 53BP1 via ATM, which is accompanied by the recruitment of the RIF1 factor. This process causes dissociation between 53BP1 and TIRR (Tudor interacting repair regulator) proteins. TIRR depletion changes a soluble fraction of the 53BP1 protein, which leads to destabilization of the NHEJ repair pathway. These data showed that the function of 53BP1 is affected by many factors, including histone post-translational modifications. For example, DNA damage-specific H4K16 acetylation disrupts the binding of the 53BP1 protein to H4K20 methylation. On the other hand, the histone deacetylases HDAC1 and HDAC2 deacetylate H4K16 upon genome injury, which potentiates an interaction between 53BP1 and H4K20me1/me2 [18,22]. The function of the 53BP1 protein in damaged chromatin is also regulated via histone H2A ubiquitination at the position of lysine 15 (H2AK15ub), which is mediated by the ubiquitin ligase RNF168 [23]. All of these results show a crosstalk between the specific histone signature and the DDR-related function of the 53BP1 protein.

H3K9 tri-methylation is also a very attractive histone modification, discussed from the view of the DNA damage response [24]. In a physiological state of the cell, this type of post-translational histone modification contributes to the formation of heterochromatin, which is abundant on HP1 protein isoforms. In particular, H3K9me3, together with HP1α and HP1β proteins, stabilizes heterochromatin and plays a role in DNA damage response. For example, Ayrapetov et al. [25] showed that the H3K9me3 level regulates activation of ATM kinase, but the direct role of H3K9 methylation in chromatin with DNA lesions is not completely clear. Ayrapetov et al. [25] documented that Suv39h1 HMT is rapidly recruited to DNA lesions, where this enzyme regulates H3K9 methylation in the vicinity of double-strand breaks. Moreover, loss of inducible H3K9me3 at DNA lesions was found to be associated with non-physiological DNA repair. Interestingly, the transient release of the Kap-1/HP1/Suv39h1 complex from chromatin appeared when Kap-1 was phosphorylated by ATM. Sun et al. [26] showed that the DNA repair complex Mre11-Rad50-Nbs1 targets Tip60 histone acetyltransferase to H3K9me3. This nuclear event likely implies a very important functional role of H3K9me3 in homologous recombination repair (HRR) [25].

Here, we were inspired by the fact that H3K9 tri-methylation, as the HP1 binding partner, takes part in the DNA damage response. We have studied the DNA repair-related function of Suv39h1/h2 enzymes, which are responsible for H3K9me3. We analyzed the effect of the Suv39h1/h2 point mutation or deletion on protein recruitment to DSBs, and we studied the epigenetics of locally micro-irradiated chromatin. We analyzed whether Suv39h1/h2 depletion could change H3K9me3 and H3K9ac or the recruitment of HP1β and 53BP1 proteins to locally irradiated chromatin. Our main question was, to what extent is H4K20me1/me2/me3 changed upon DNA injury, and how does depletion in Suv39h1/h2 HMTs affect the function of H4K20 methylation at DNA lesions? In addition, we analyzed whether H4K20 tri-methylation is recognized by the 53BP1 protein recruited to DSB sites, and whether H4K20me3 contributes to the NHEJ repair pathway to a similar extent as H4K20me2.

## RESULTS

### The Suv39h1/h2-dependent link between H3K9 methylation and H4K20 tri-methylation

**Figure 1:**
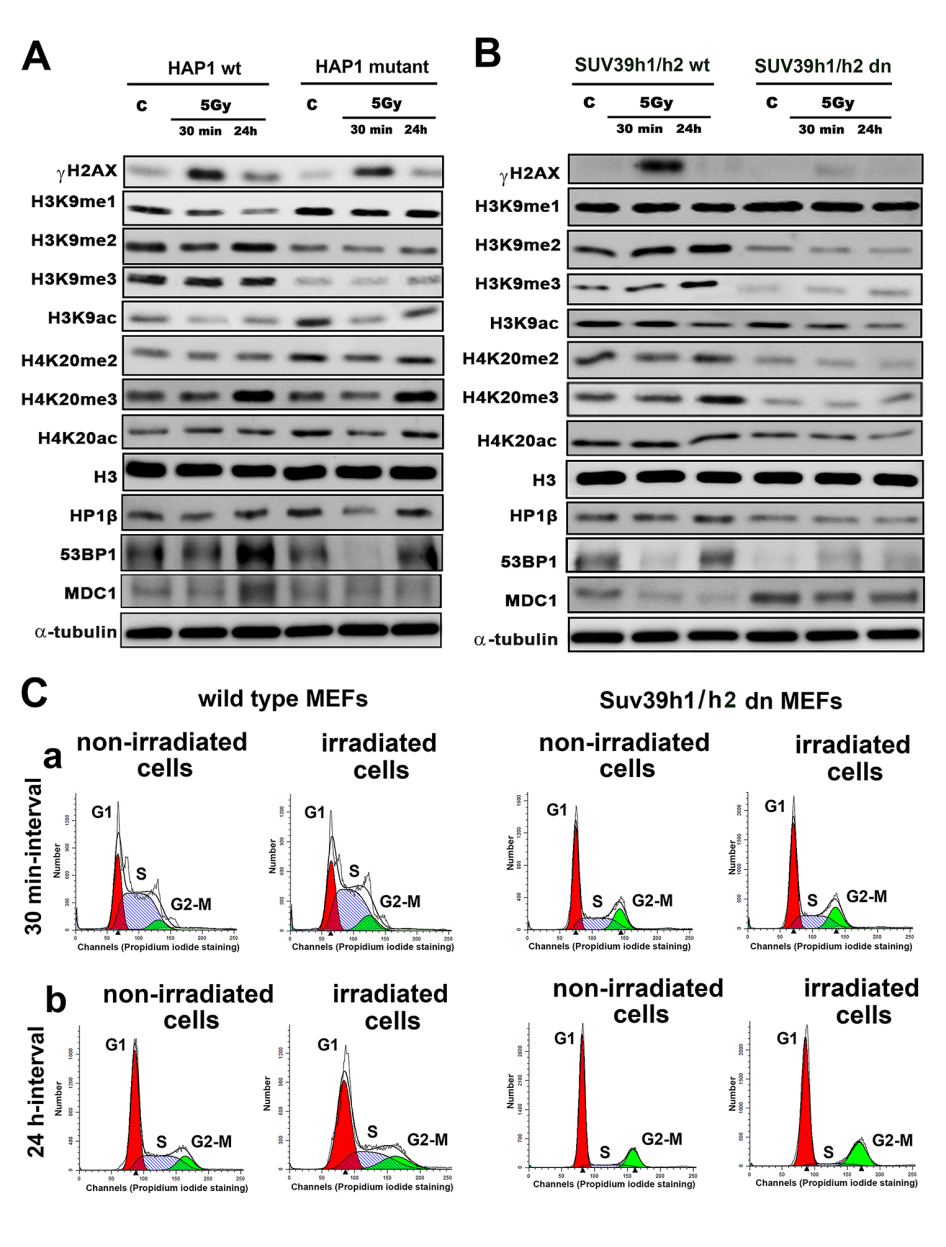
**Histone signature in non-irradiated and irradiated cells without and with mutation or deletion in Suv39h1/h2 histone methyltransferases.** Western blot analysis of γH2AX, H3K9me1, H3K9me2, H3K9me3, H3K9ac, H4K20me2, H4K20me3 and H4K20 acetylation. The levels of modified histones were normalized to that of total H3 histones. As DNA damage markers, 53BP1, MDC1 proteins, and the HP1β protein were studied and normalized to the level of α-tubulin. Protein levels were studied in (**A**) HAP1 wt and HAP1 cells with the mutation in the SUV39H1 gene and (**B**) in wt MEFs and Suv39h1/h2-deficient fibroblasts (MEFs). Non-irradiated cells and cells irradiated by 5 Gy of γ-rays (harvested 30 min and 24 hours after irradiation) were analyzed. (**C**) Cell cycle profiles were studied by flow cytometry in non-irradiated and γ-irradiated wt MEFs and non-irradiated and γ-irradiated Suv39h1/h2 dn mouse embryonic fibroblasts. Panel (**a**) shows a 30-min interval, and panel (**b**) shows a 24-h interval when the cells were harvested after irradiation (and related control samples). Using Mod-Fit software, the percentage of cells in G1 (red peak), S (dash blue peak) and G2-M (green peak) cell cycle phases was calculated. The average cell cycle profile is shown for individual samples, and experiments were performed in 3 biological replicates.

Here, we show how γ-irradiation changes the histone signature in human and mouse cells, as well as in histone post-translation modifications in human cells with the mutation in the SUV39H1 gene or mouse embryonic fibroblasts (MEFs) with an abrogated function of histone methyltransferases Suv39h1/h2. We analyzed the following histone markers: γH2AX, H3K9me1, H3K9me2, H3K9me3, H3K9ac, H4K20me2, H4K20me3, H4K20ac and markers of DNA lesions 53BP1 and MDC1 proteins. We confirmed that H3K9me3 is reduced in HAP1-SUV39H1 mutant cells, and we observed a low level of H3K9me3 in Suv39h1/h2 dn MEFs, which was characterized by an absence of H3K9me3 at chromocenters (Fig. 1A, B, 2A, B) [27].

**Figure 2:**
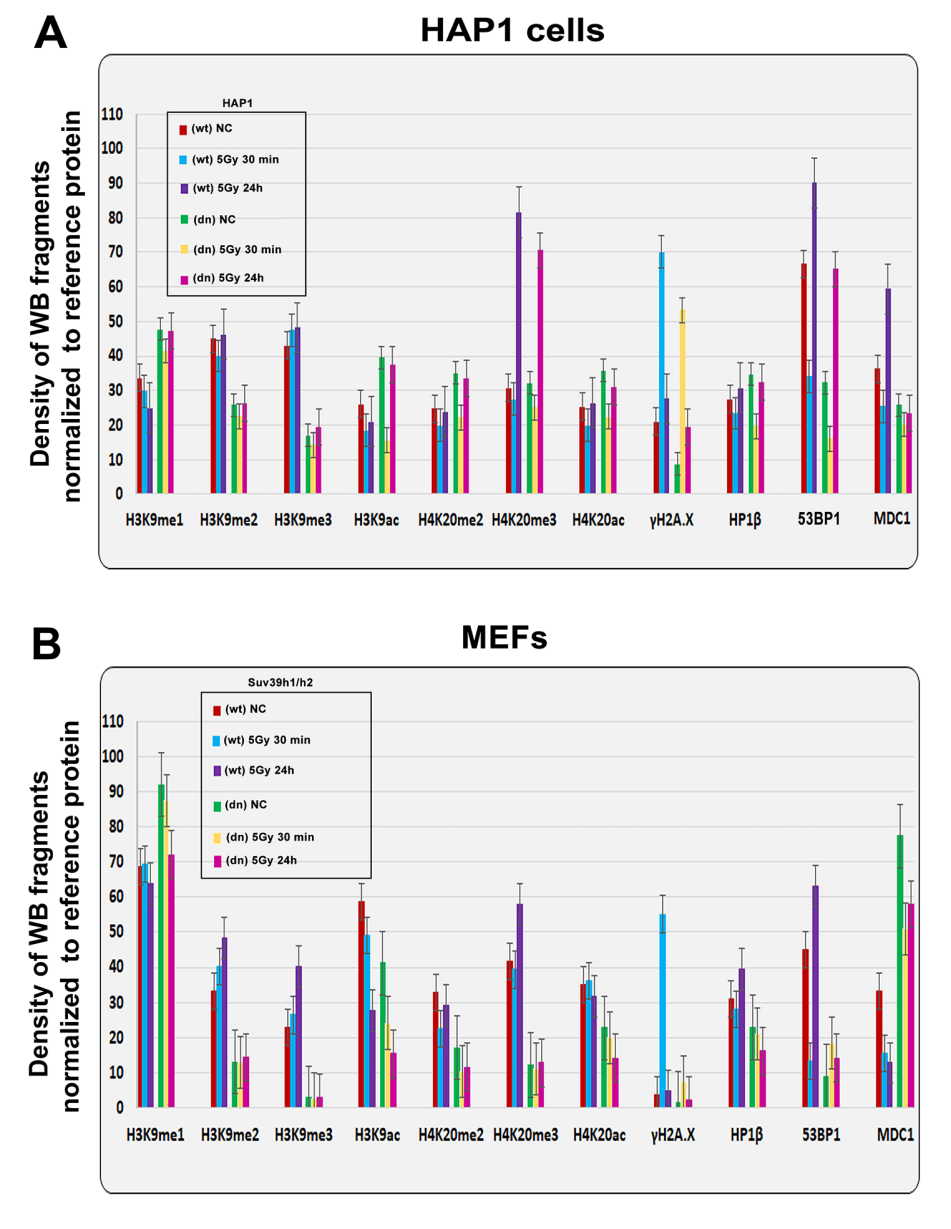
**Quantification of western blot data on selected histone markers in non-irradiated and γ-irradiated cells.** Using ImageJ and ImageQuant TL software, the levels of the following proteins (originated from Fig. 1A, B) were quantified: H3K9me1, H3K9me2, H3K9me3, H3K9ac, H4K20me2, H4K20me3, H4K20ac, γH2AX, HP1β, 53BP1, and MDC1. The levels of modified histones were normalized to those of total H3 histones, and those of DDR-related proteins were normalized to those of α-tubulin. Quantification was performed in the following samples: (**A**) HAP1 wt and HAP1 mutant cells and (**B**) wt MEFs and Suv39h1/h2-deficient fibroblasts (MEFs).

We also verified the effect of radiation on the histone signature. We found that levels of the well-known DNA repair marker γH2AX were increased 30 min after cell exposure to γ-radiation in the HAP1 wt, HAP1 mutant and in Suv39h1/h2 wt cells (Fig. 1A, B, 2A, B). However, an increase in γH2AX after irradiation was not pronounced in Suv39h1/h2 dn MEFs (Fig. 1B, quantification Fig. 2B). Interestingly, 24 hours after γ-irradiation, γH2AX had decreased in all cell types studied compared with the interval of 30 min after irradiation (Fig. 1A, B, 2A, B). The described changes in the γH2AX levels at the 30-min interval were not affected by cell cycle changes, which were potentially caused by irradiation (Fig. 1 Ca). Together, in the 30-min interval, cell cycle profiles were nearly identical in non-irradiated and γ-irradiated cells when we compared Suv39h1/h2 wt versus Suv39h1/h2 dn MEFs (Fig. 1 Ca). A non-significant cell shift towards G2-M was found in the 24-hour interval compared with non-irradiated and γ-irradiated cell populations (Fig. 1 Cb). We observed the following percentage of cells in individual cell cycle phases: non-irradiated wt cells: G1=56.11±1.9%; S=31.39±1.9% and G2-M=12.50±0.1%; irradiated wt cells: were characterized by following cell cycle profile: G1=53.30±0.19%; S=30.10±1.7% and G2-M=16.6±1.5%; non-irradiated Suv39h1/h2 dn cells: 69.50±1.2%; 11.96±2.3% and 18.54±1.2%; irradiated Suv39h1/h2 dn cells: G1=68.75±0.6%; S=7.10±0.9% and G2-M=24.15±0.9% of the cells. In this case, an increase in γH2AX that was observed 30 min after irradiation was unlikely to be affected by the accumulation of cells in G2, which is characterized by genome duplication and can appear when the cells are exposed to γ-rays [5].

From the view of the histone signature in Suv39h1/h2 dn MEFs, compared with the wt counterpart, we observed not only a low level of H3K9me3 but also a decrease in H3K9me2. Western blots showed, in the majority of cases, no significant irradiation-induced changes in H3K9me1/me2/me3 in HAP1 wt, HAP1 mutant or Suv39h1/h2 wt and Suv39h1/h2 dn cells (Fig. 1A, B and 2A, B). In these experimental systems, the levels of H4K20me2/me3 were reduced, but H3K9me1, H3K9ac, and H4K20ac levels were not changed when we compared Suv39h1/h2 dn fibroblasts with wt MEFs (Fig. 1B, 2B). These results implied a Suv39h1/h2-dependent link between H3K9me2/me3 and H4K20me2/me3. Thus, we further tested whether depletion of Suv39h1/h2 methyl transferases affects 53BP1-dependent DNA damage response mediated via H3K9me3 and H4K20me2/me3. For the majority of experiments, we selected Suv39h1/h2 wt and Suv39h1/h dn cells because non-irradiated control cells are characterized by a very low level of γH2AX, which is important for studies on radiation effects that induce a pronounced phosphorylation of histone H2AX.

### Nuclear distribution pattern of the 53BP1 protein that binds to H4K20 methylation

**Figure 3:**
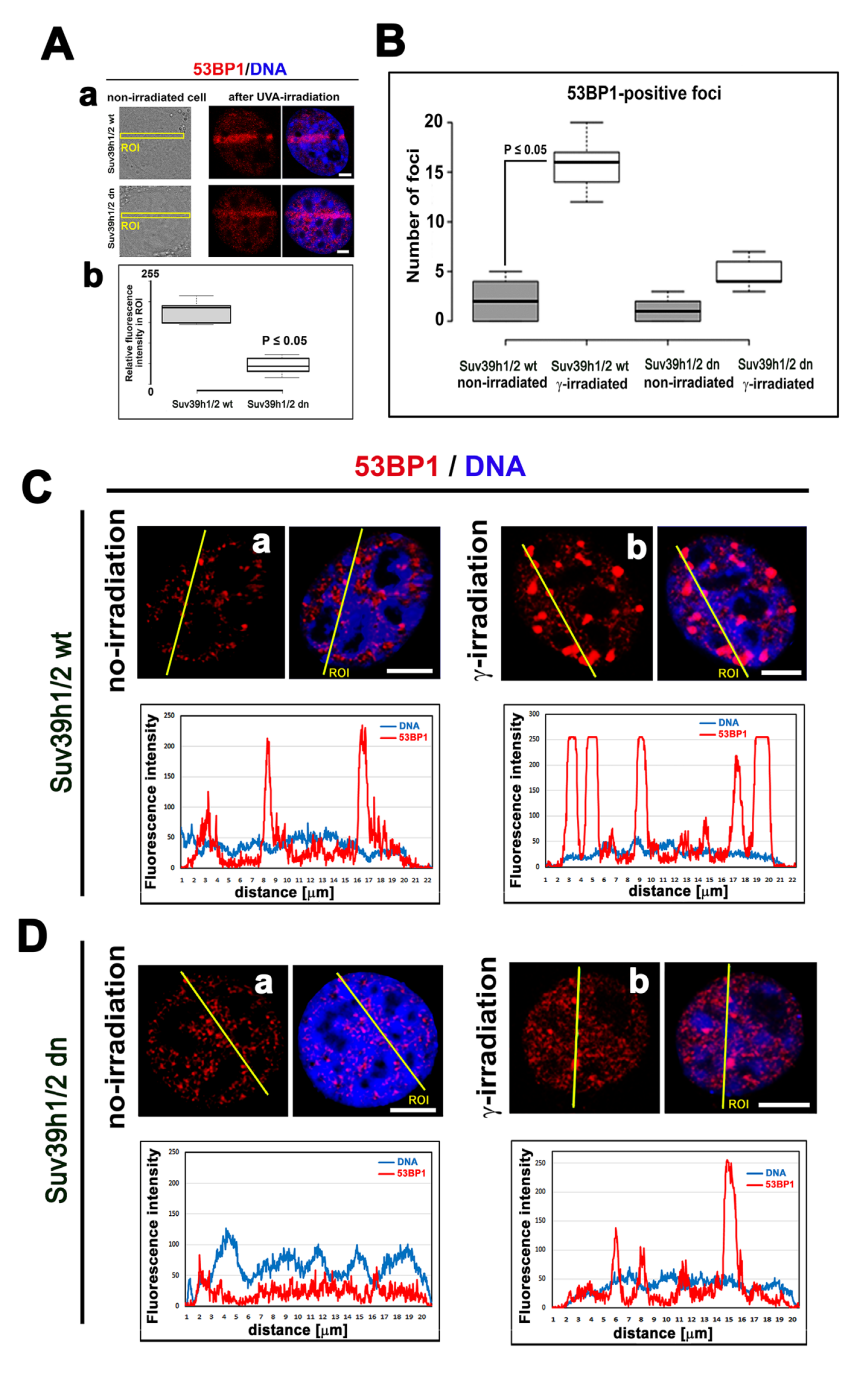
**The nuclear distribution pattern of 53BP1 protein.** (**A**) Recruitment of 53BP1 to locally micro-irradiated chromatin in (**a**) Suv39h1/h2 wt and (**b**) Suv39h1/h2 dn fibroblasts. Quantification using LAS AF software in panel (b) shows that the level of the 53BP1 protein at UVA-irradiated ROIs was reduced in Suv39h1/h2 dn MEFs. For statistical analysis, the Student’s t-test was used; the difference in the protein levels is statistically significant at P≤0.05. (**B**) An average number of 53BP1-positive foci was significantly increased in γ-irradiated Suv39h1/h2 wt MEFs compared with those in the non-irradiated wt counterpart. In Suv39h1/h2 dn MEFs, γ-irradiation did not significantly change a number of 53BP1-positive repair foci. Student’s t-test was used for statistical analysis, and statistical significance was defined as P≤0.05. The distribution of 53BP1 in (**C**) in (**a**) non-irradiated and (**b**) γ-irradiated wild-type MEFs and (**D**) (**a**) non-irradiated and (**b**) γ-irradiated Suv39h1/h2 dn MEFs. Scale bars represent 10 µm.

The function of H4K20 methylation contributes to the DNA damage-related function of the 53BP1 protein. Thus, we also studied the nuclear distribution pattern of the 53BP1 protein. In Suv39h1/h2 dn cells (in comparison to the wt counterpart), we showed that the level of 53BP1 is significantly decreased (at p≤0.05) in UVA-irradiated ROIs (Fig. 3 Aa, b). At the global cellular level, we observed a well-known phenomenon linked to the number and morphology of 53BP1-positive spontaneous foci and γ-irradiation-induced foci (IRIF). We found that the formation (an increased number) of IRIF was statistically significant in irradiated Suv39h1/h2 wt cells when compared to non-irradiated counterparts. However, Suv39h1/h2 depletion prevented, to some extent, to the formation of 53BP1-positive IRIF (Fig. 3B, Ca, b, Da, b). Considering the protein levels, the 53BP1 level was decreased in HAP1 mutant cells and Suv39h1/h2 wt fibroblasts, 30 min after global irradiation by γ-rays, while 24 h after cell exposure to γ-irradiation, the level of 53BP1 was recovered to the original level, which was observed before irradiation (Fig. 1A, B). On the other hand, Suv39h1/h2 dn cells were characterized by an increase in 53BP1 protein levels 30 min after γ-irradiation; 24 h after cell exposure to γ-rays, the level of 53BP1 was decreased to the original level, as observed in non-irradiated Suv39h1/h2 dn cells (Fig. 1B, 2B). In general, in Suv39h1/2 dn cells, the level of the 53BP1 protein was relatively low (Fig. 1B).

### A decrease in H3K9 acetylation appears at micro-irradiated chromatin, but H3K9ac at γH2AX-positive lesions was not affected by Suv39h1/h2 depletion

**Figure 4:**
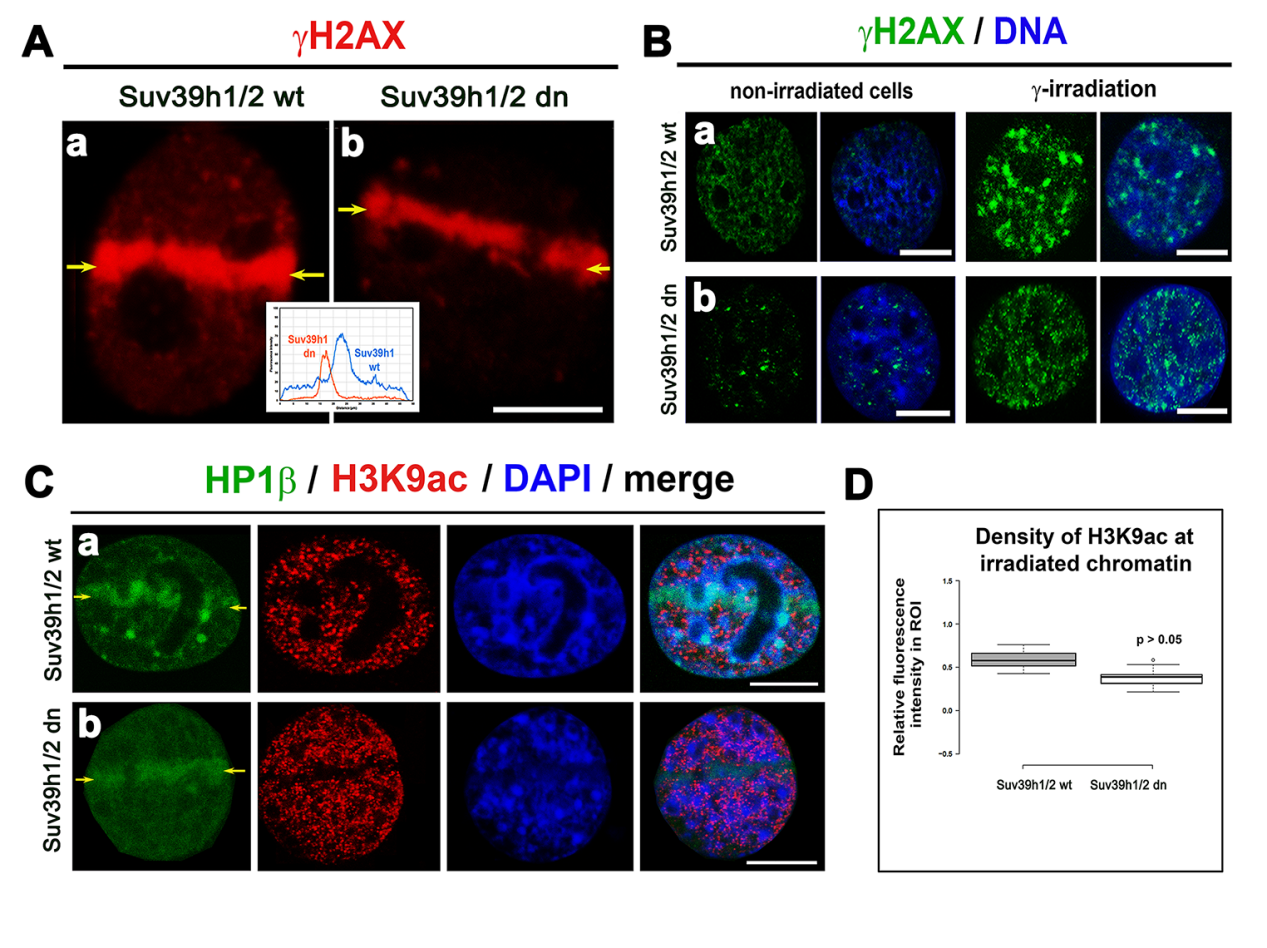
**The level of γH2AX in irradiated chromatin. Recruitment of HP1β protein at γH2AX-positive DNA lesions and H3K9 deacetylation in UV-damaged chromatin.** (**A**) Analysis of the level of γH2AX (red) in (a) Suv39h1/h2 wt and (b) Suv39h1/h2-deficient fibroblasts (MEFs). The appearance of γH2AX (red) was studied in micro-irradiated ROIs (see yellow arrows) induced by UVA laser (355 mm). (**B**) Levels of γH2AX (green) in non-irradiated and γ-irradiated (a) Suv39h1/h2 wt and (b) Suv39h1/h2 dn MEFs. Whole cell populations were irradiated by γ-rays. DAPI (blue) was used as a counterstain. Scale bars represent 10 µm. (**C**) Accumulation of HP1β (green) and the level of H3K9 deacetylation (red) in locally micro-irradiated (a) Suv39h1/h2 wt and (b) Suv39h1/h2 dn MEFs. DAPI was used as a counterstain of a whole nuclear volume. (**D**) Quantification of H3K9ac (red) in micro-irradiated chromatin showed an identical decrease of H3K9ac in micro-irradiated regions of interest (ROIs shown by yellow arrows in panels (Ca, b). Studies were performed in locally micro-irradiated Suv39h1/h2 wt and Suv39h1/h2 dn MEFs. Scale bars are 10 µm.

Prior to an analysis of H3K9 acetylation in radiation-damaged chromatin, we analyzed the level of γH2AX in UVA-micro-irradiated cells and in cell populations exposed to γ-rays. We observed that in the UVA micro-irradiated region of Suv39h1/h2 dn cells, the γH2AX level was decreased compared with Suv39h1/h2 wt fibroblasts, which was characterized by a pronounced phosphorylation of H2AX at UVA-induced DNA lesions (Fig. 4 Aa, b). Irradiation by γ-rays increased γH2AX more significantly in Suv39h1/h2 wt cells than in Suv39h1/h2 dn fibroblasts (Fig. 1B and 4 Ba, b).

**Figure 5:**
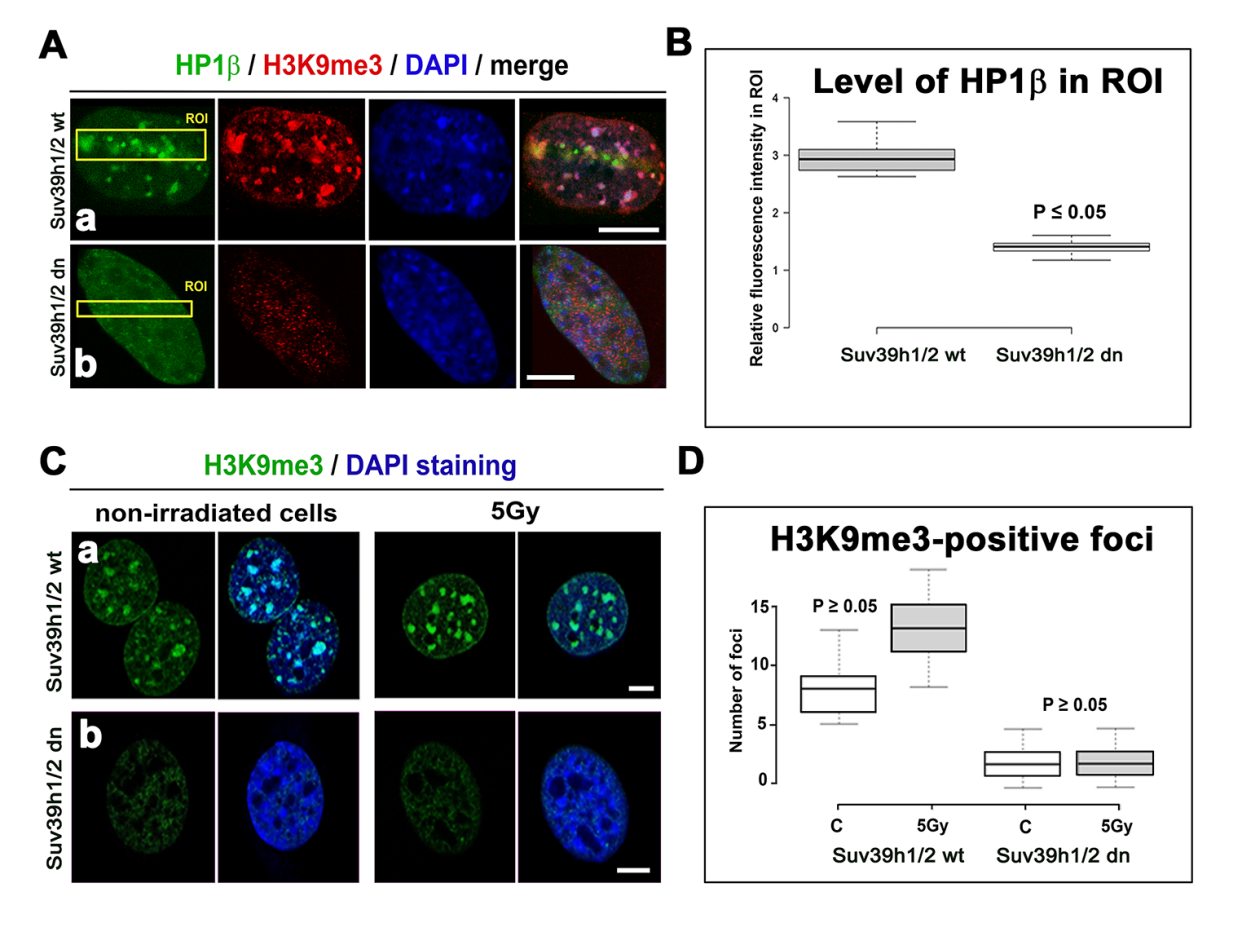
**Accumulation of HP1β and H3K9 tri-methylation (red) in locally micro-irradiated genomic region of Suv39h1/h2 wt and Suv39h1/h2 dn MEFs.** (**Aa**) The fluorescence of GFP-tagged HP1β (green) was decreased in regions of interest (ROIs are shown by yellow rectangles) in Suv39h1/h2 dn cells compared with the wt counterpart. The level of H3K9me3 was high in the chromocenters of locally micro-irradiated wt cells. H3K9me3 was very low in Suv39h1/h2 dn cells. (**Ab**) A decrease in GFP-tagged HP1β in irradiated ROI of Suv39h1/h2 dn cells. (**B**) Quantification of HP1β in UVA-irradiated ROI in Suv39h1/h2 wt and Suv39h1/h2 dn cells. Difference in the fluorescence intensity in ROI was statistically significant as shown by statistical analysis at P≤0.05 (Student’s t-test). (**C**) Nuclear distribution pattern of H3K9me3 (green) in non-irradiated and γ-irradiated Suv39h1/h2 wt and Suv39h1/h2 dn MEFs. Wild-type MEFs were characterized by H3K9me3-positivity in chromocenters (clusters of centromeric heterochromatin), while H3K9me3 was low in nuclei of Suv39h1/h2 dn cells. Scale bars are 10 µm. (**D**) Quantification of H3K9me3-positive foci in non-irradiated and γ-irradiated Suv39h1/h2 wt and Suv39h1/h2 dn MEFs. Irradiation by γ-rays did not significantly affect a number of H3K9me-positive foci, as shown by statistical analysis (P≥0.05); Student’s t-test was used.

A radiation-induced increase in γH2AX was accompanied by H3K9 deacetylation, which we found at the locally micro-irradiated chromatin of both wt and Suv39h1/h2 dn cells (Fig. 4 Ca, b; D). In Suv39h1/h2 dn cells, this epigenetic profile was accompanied by a weakened accumulation of HP1β to locally induced DNA lesions when compared with Suv39h1/h2 wt MEFs (Fig. 4 Ca, b). This phenomenon was likely to be associated with the depletion of H3K9me3 in Suv39h1/h2 dn fibroblasts (Fig. 1B, 5 Aa, b; B). However, in Suv39h1/h2 wt cells, the H3K9me3 was high, especially at locally micro-irradiated chromo-centers (clusters of centromeric heterochromatin) that were positive for HP1β (Fig. 5 Aa) [27]. The number of H3K9me3-positive foci in wt MEFs was increased by γ-irradiation of the whole cell population, but data were not statistically significant. As expected, chromocenters of Suv39h1/h2 dn cells did not contain H3K9me3, and this histone distribution pattern was not changed by γ-irradiation (Fig. 5 Ca, b, D).

### Irradiated cells by γ-rays are characterized by an increase in the level of H4K20me3 but not H4K20me1/me2, and Suv39h1/h2 depletion reduces a pronounced H4K20me3 at micro-irradiated chromatin

By western blot analysis, we observed that H4K20me2/me3 levels were reduced in Suv39h1/h2-depleted fibroblasts compared to wt cells. In comparison to non-irradiated cells, γ-irradiation did not change the H4K20me2 in HAP1 wt, HAP1 mutant, Suv39h1/h2 wt and Suv39h1/h2 dn cells (Fig. 1A, B). On the other hand, H4K20me3 was increased 24 hours after γ-irradiation in HAP1 wt, HAP1 mutant cells, and Suv39h1/h2 wt fibroblasts (Fig. 1A and 2A).

**Figure 6:**
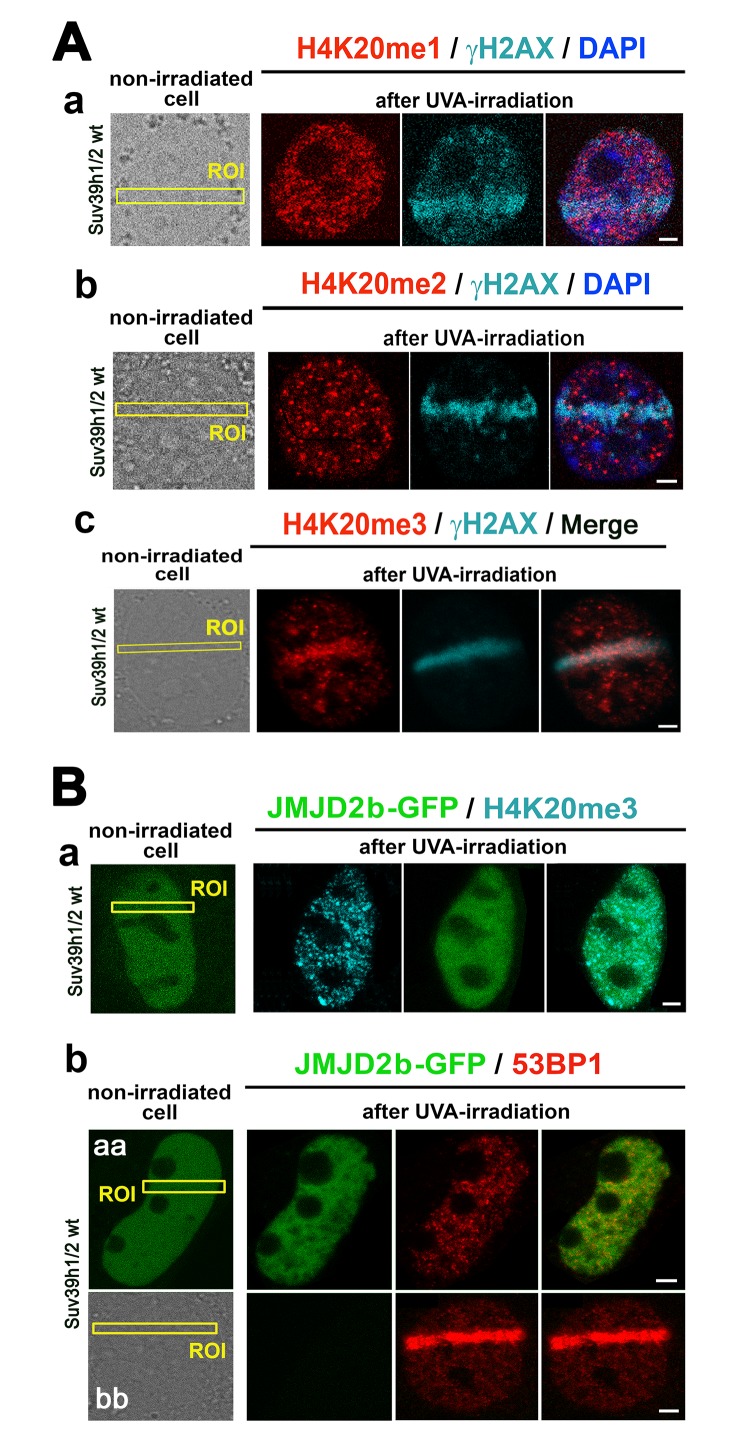
**The nuclear distribution pattern of H4K20me1/me2/me3 at DNA lesions.** (**A**) The levels of (**a**) H4K20me1 (red) in γH2AX-positive DNA lesions (magenta), (**b**) H4K20me2 (red) in γH2AX-positive DNA lesions (magenta), and (**c**) H4K20me3 (red) in DNA lesions studied in parallel with γH2AX (magenta). (**B**) The level of (**a**) H4K20me3 and (**b**) 53BP1 in micro-irradiated ROI of the cells over-expressing JMJD2b histone demethylase, tagged by GFP. Panels Bb-aa show low level of 53BP1 at micro-irradiation induced DNA lesions in cells over-expressing GFP-tagged JMJD2b and panel Bb-bb documents 53BP1 recruitment to DSB sites in the cells with a normal expression of JMJD2b. Scale bars in all panels represent 5 µm.

By local laser micro-irradiation, we observed that the levels of both H4K20me1 and H4K20me2 were identical at micro-irradiated chromatin in comparison with the surrounding genome, while a pronounced H4K20me3 appeared at γH2AX-positive DNA lesions 8-10 min after laser exposure (Fig. 6 Aa-c). This DDR-related nuclear event was not only dependent on Suv39h1/h2 function but also linked to H3K9me3, because when we over-expressed JMJD2b histone demethylase, antagonizing H3K9me2/me3 in the pericentromeric heterochromatin [28], the H4K20me3 level at DNA lesions was not increased (Fig. 6 Ba). Similarly, in the cells over-expressing GFP-JMJD2b, the 53BP1 protein was not recruited to micro-irradiated chromatin (Fig. 6 Bb - aa). However, in the cells, in which we did not over-expressed JMJD2b, the level of the 53BP1 protein at micro-irradiated genomic region was pronouncedly increased (Fig. 6 Bb - bb).

**Figure 7:**
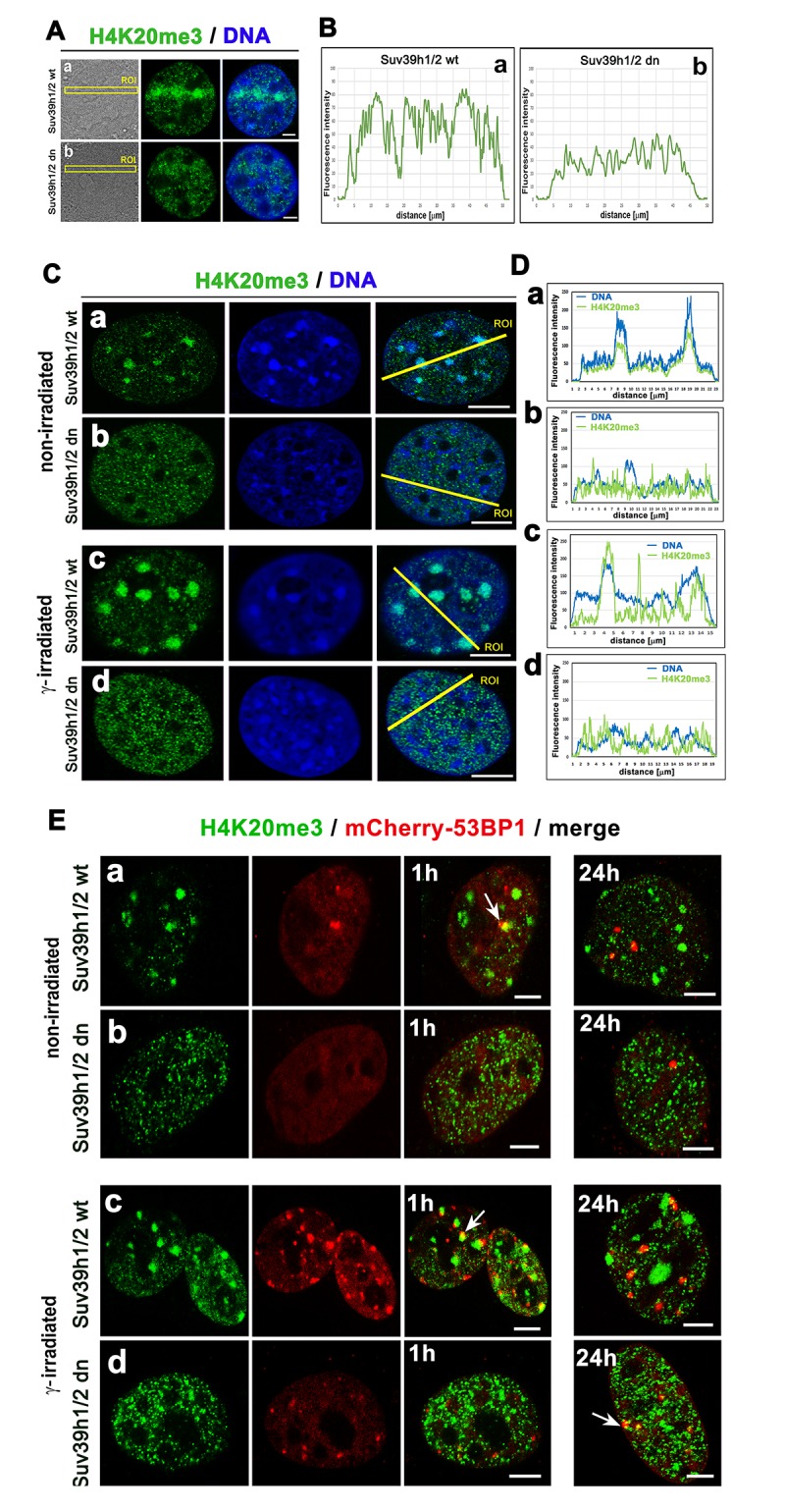
**The nuclear distribution pattern of H4K20me3 (green) in UV-damaged chromatin**. (**Aa**) Non-irradiated and γ-irradiated Suv39h1/h2 wt and (**Ab**) non-irradiated and γ-irradiated Suv39h1/h2 dn MEFs. DAPI (blue) was used as a counterstain. Scale bars represent 10 µm. (**B**) Quantification of H4K20me3 (green) shown in panels Aa, Ab. Quantification of H4K20me3 was performed according to the selected region of interests (ROIs, yellow lines). LAS AX software was used for analysis of fluorescence intensities. (**C**) Nuclear distribution pattern of H4K20me3 in (**a**) non-irradiated and (**c**) γ-irradiated Suv39h1/h2 wt and (**b**) non-irradiated and (**d**) γ-irradiated Suv39h1/h2 dn fibroblasts. (**D**) Quantification of fluorescence intensity of H4K20me3 is shown in panel Ca-d. Analysis by LAS AF software was performed in (**a**) non-irradiated and (**c**) γ-irradiated Suv39h1/h2 wt and (**b**) non-irradiated and (**d**) γ-irradiated Suv39h1/h2 dn MEFs. (**E**) Nuclear distribution of H4K20me3 (green) and the 53BP1 protein (red) in (**a**) non-irradiated and (**c**) γ-irradiated Suv39h1/h2 wt and (**b**) non-irradiated and (**d**) γ-irradiated Suv39h1/h2 dn cells.

Considering the nuclear distribution pattern of H4K20me3, we observed an increase in this histone mark in the nucleoplasm of micro-irradiated Suv39h1/h2 wt cells (Fig. 7 Aa, Ba), while H4K20me3 in micro-irradiated chromatin of Suv39h1/h2 dn cells was remarkably low (Fig. 7 Ab, Bb). In non-irradiated wt cells, H4K20me3 was highly positive in chromocenters that become robust after cell exposure to γ-rays (Fig. 7 Ca, Cc). However, the formation of chromocenters was abrogated in Suv39h1/h2 dn cells; thus, low level of H4K20me3, similar to H3K9me3, was homogeneously dispersed through the nucleoplasm, and γ-irradiation did not affect the nuclear distribution pattern of H4K20me3 in these cells (Fig. 5 Ab; 7 Cb, d; Da-d). To this information, we found H4K20me3 positivity in 53BP1-positive spontaneous DNA lesions and IRIF in both wt and Suv39h1/h2 dn cells (Fig. 7 Ea-d).

### Cell-cycle-dependent pronounced H4K20me3 at micro-irradiated chromatin

**Figure 8:**
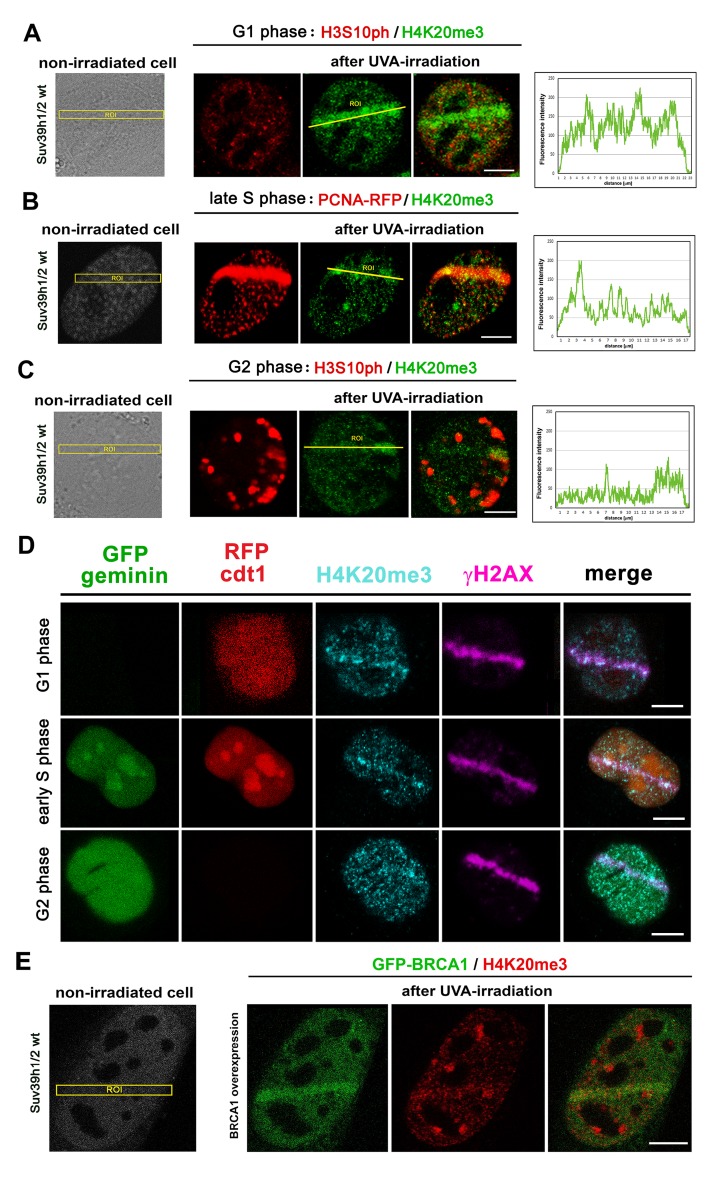
**The level of H4K20me3 in cell cycle phases.** H4K20me3 in (**A**) G1, (**B**) S, and (**C**) G2 phases of the cell cycle. G1 and G2 phases were determined according to the nuclear distribution pattern of H3S10 phosphorylation: the level of H3S10p is low in the G1 phase characterized by an appearance of tiny H3S10p-positive signals. G2 phase is characterized by robust H3S10p-positive signals in the cell nucleus. The S phase was recognized according to the distribution pattern of the mCherry-tagged PCNA protein that appeared at DNA lesions in the late S phase. The highest level of H4K20me3 at DNA lesions was in the G1 phase of the cell cycle, while cells in S and G2 phases of the cell cycle were characterized by reduced H4K20me3 at micro-irradiated chromatin. Scale bars represent 10 µm. (**D**) The phenomenon observed in panels A-C was confirmed by the use of the HeLa-Fucci cellular system showing G1 cells expressing RFP-cdt1 and G2 cells with GFP-geminin positivity. The cells in early S phase were subtly positive for both RFP-cdt1 and GFP-geminin. (**E**) The level of H4K20me3 (red) in mouse fibroblast over-expressing GFP-tagged BRCA1 protein (green). Scale bars in all panels represent 10 µm.

To learn more about the function of H4K20me3 at DNA lesions, we studied the level of H4K20me3 in G1, S, and G2 phases of the cell cycle. We recognized G1 and G2 phases according to the nuclear distribution pattern of H3S10 phosphorylation [29] and S phase according to the distribution pattern of proliferating cell nuclear antigen (PCNA), which recognizes DNA lesions in late S and G2 phases of the cell cycle [30]. In the G1 phase of the cell cycle, we found the highest level of H4K20me3 at DNA lesions; however, in the G2 phase, we observed a lower density of H4K20me3 at locally micro-irradiated chromatin (Fig. 8A-C). These data fit well the observation that H4K20me2 is involved in the NHEJ repair pathway mediated via the 53BP1 protein, and we observed this phenomenon for H4K20me3 (Fig. 6A, B). This claim we also confirmed by the use of the Fucci cellular system expressing RFP-tagged cdt1 in the G1 phase and GFP-tagged geminin in the G2 phase of the cell cycle. In these cells, the early S phase was recognized by the subtle expression of both RFP-cdt1 and GFP-geminin (Fig. 8D). By the use of HeLa-Fucci cells, we verified that pronounced H4K20me3 appears at 53BP1-positive DNA lesions of G1 cells. When we experimentally induced BRCA1 over-expression; thus, we mimicked the situation at DNA lesions that likely appear in the G2 phase of the cell cycle, these cells were characterized by a very low level of H4K20me3 at micro-irradiated chromatin (Fig. 8E).

### Immunoprecipitation shows an interaction between 53BP1 and H3K9me3 or 53BP1 and H4K20me2/me3, but not 53BP1 and HP1β proteins

**Figure 9:**
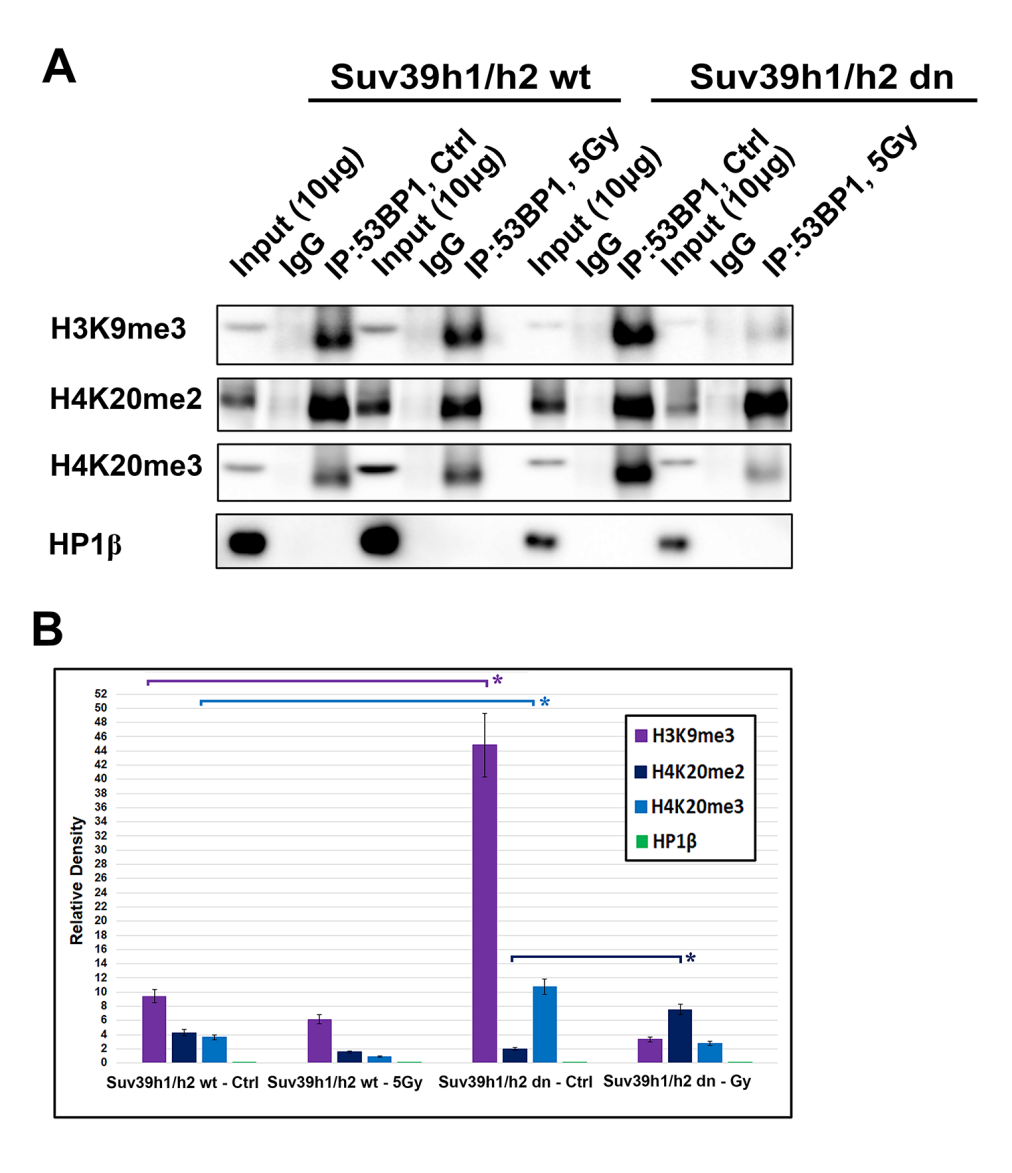
**An interaction between 53BP1-H4K20me2/me3.** (**A**) Immunoprecipitation (IP) experiments showed an interaction between H3K9me3 and 53BP1 or H4K20me2 and 53BP1 or H4K20me3 and the 53BP1 protein. HP1β protein did not interact with the 53BP1 protein. (**B**) Quantification of IP fragments from panel (A) studied in non-irradiated and γ-irradiated Suv39h1/h2 wt and Suv39h1/h2 dn cells. Asterisks (*) indicate statistical significance at P≤0.05.

Immunoprecipitation experiments revealed an interaction between H3K9me3 and 53BP1 or H4K20me2 and 53BP1 or H4K20me3 and 53BP1 proteins (Fig. 9A). Interestingly, mutual interaction between H3K9me3 and 53BP1 or H4K20me3 and 53BP1 was enhanced in Suv39h1/h2 dn cells when compared with the wt counterpart (Fig. 9B). In addition, γ-irradiation enhanced the interaction between H4K20me2 and 53BP1 in Suv39h1/h2 dn cells when compared to non-irradiated dn fibroblasts (Fig. 9A, and quantification in Fig. 9B).

The HP1β protein, as a binding partner of H3K9me3, did not interact with the 53BP1 protein (Fig. 9A, B). Thus, considering the DNA repair pathways, H3K9me3 and H4K20me2/me3, but not HP1β, could be important epigenetic factors that contribute to the regulation of the 53BP1-dependent DNA repair pathway. The function of HP1β in DNA repair was not dependent on H3K9me3; therefore, the HP1β protein is unlikely to be involved in the NHEJ mechanism. This observation agrees with Luijsterburg et al. [31], showing that HP1 isoforms recognize UV-induced DNA lesions irrespective of its CD domain recognizing H3K9me3. This claim fits well with HP1β appearing in damaged genomic regions as positive on cyclobutene pyrimidine dimers (CPDs). Thus, HP1β likely plays a role in the nucleotide excision repair (NER) mechanism [32], but H3K9me3 and H4K20me3 contribute to the function of 53BP1-mediated NHEJ repair.

## DISCUSSION

In general, specific histone post-translational modifications are recognized by proteins of DNA repair signaling pathways [33]. Histone-related epigenetic features regulate the DNA damage response, and we showed here that, conversely to H4K20me1 and H4K20me2, a pronounced H4K20 tri-methylation appears at DNA lesions induced by local micro-irradiation (Fig. 6 Aa-c). This epigenetic process was most pronounced in the G1 phase of the cell cycle; moreover, a depletion of histone methyltransferases Suv39h1/h2 prevented a pronounced H4K20me3 at DSB sites. From the view of the DNA damage response, the most attention was dedicated to H4K20 di-methylation, which represents a binding epigenetic marker for the 53BP1 protein in DNA repair [11,12]. However, here, we showed that the H4K20me2 level was not changed in irradiated chromatin (Fig. 1A, B and 6 Ab). However, we cannot rule out that the level of H4K20me2 at micro-irradiated genomic regions is not sufficient for the binding of the 53BP1 protein to DNA lesions, as was published by other authors (ref. 11, 12 and shown in Fig. 6 Ab). On the other hand, little is known about the function of H4K20me3 in DNA repair. Up to now, several studies on gene regulation showed that H4K20me3 is rather essential in the process of gene silencing. On the other hand, H4K20me2 was found not to be involved in the regulation of transcription mediated via the 53BP1-p53 signaling pathway [12,13].

It is well known that after DNA damage, 53BP1 binds to H4K20me2 via the 53BP1 tandem Tudor domain. Hsiao and Mizzen [34] showed that 53BP1 foci are formed near histones H4, lysine 20 positions that are di-methylated by Suv4-20h or SETD8 HMTs prior to DNA damage. Here, we document that, compared with H4K20me2, H4K20me3 was more significantly changed after cell exposure to γ-rays (Fig. 1A, B and  2A, B) and only pronounced H4K20me3, but no changes in H4K20me1/me2 appeared at micro-irradiated genomic regions (Fig. 6 Aa-c). We showed a mutual functional link between H4K20me3 and the 53BP1 protein (Fig. 9A, B). Moreover, H4K20me3 is essential at DNA lesions, especially in the G1 phase of the cell cycle, but when the PCNA protein appeared at damaged chromatin in very late S phase [30], H4K20me3 was reduced (Fig. 8A-D, 10A-D). In DNA repair, Simonetta et al. [20] showed that H4K20 di-methylation appears simultaneously with the MAD2L2 protein at DSB sites, which was associated with the formation of a protein complex consisting of 53BP1 and RIF1. Interestingly, non-replicating cells with a high level of H4K20me2 were characterized by a pronounced recruitment of 53BP1-RIF1-MAD2L2 to chromatin with DSBs. However, during DNA replication, when H4K20me2 was twofold diluted, the 53BP1-RIF1-MAD2L2 complex was found to be released from damaged chromatin and was replaced by the BRCA1 protein. Here, we showed that not only BRCA1 antagonizes a function of the H4K20me3-linked protein complex responsible for DDR (Fig. 8E), but the PCNA protein also is a fundamental factor making a "choice" between NHEJ and HR repair pathways, exactly in the late S phase (Fig. 8B). In this regard, another DNA repair factor, the MDC1 protein, can regulate the efficiency of HRR. In Suv39h1/h2 dn cells, which are analyzed here, we observed a high level of the MDC1 protein (Fig. 1B). In these cells, repair of DNA lesions might be preferentially mediated via the HRR mechanism.

**Figure 10:**
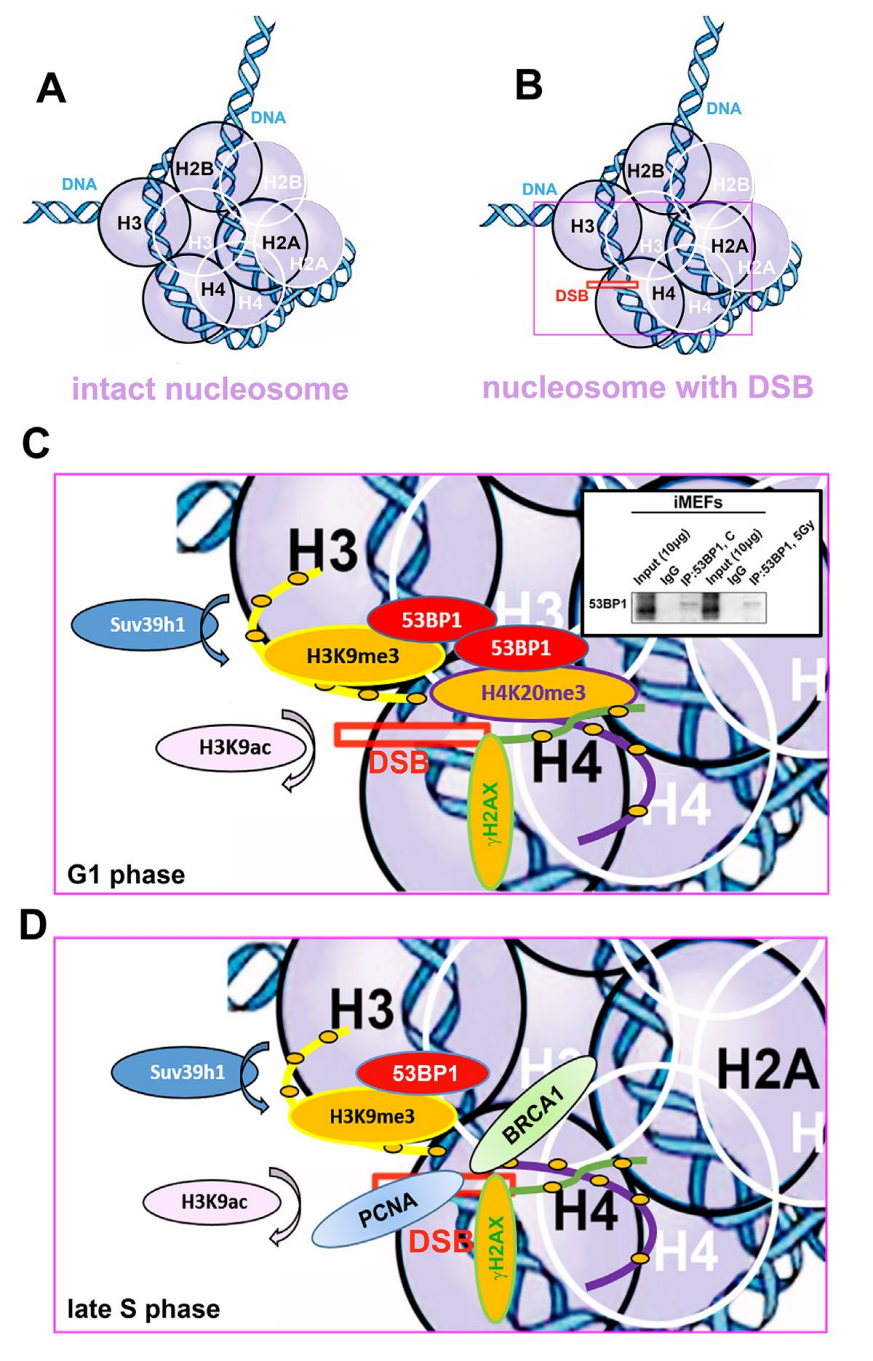
**Schematic illustration of a nucleosome with DSB occupied by studied proteins of interest in G1 and late S phase of the cell cycle.** (**A**) Schema of an intact nucleosome. (**B**) Nucleosome with wound DNA with DSB. (**C**) A pictorial illustration of histone signature and recruitment of the 53BP1 protein to DNA lesions in the G1 phase of the cell cycle. Immunoprecipitation result shows dimerization of 53BP1 protein. (**D**) An illustration of histone signature and recruitment of PCNA and BRCA1 proteins to DNA lesions in late S phase of the cell cycle. Panels represent a schematic illustration of the results.

Indeed, in Suv39h1/h2 dn cells, we observed a weakened 53BP1 functioning (level) at DSB sites; thus, repair of DNA lesions by another repair mechanism cannot be excluded (Fig. 3 Ab). However, in general, we unambiguously showed that H4K20me3 binds to the major NHEJ repair protein 53BP1 (Fig. 9A); thus, the functional relevance of H4K20me3 in G1 phase was not a surprise (Fig. 8 Aa; 10 A-C). From this view, a very important observation is that PCNA, which is recruited to DNA lesions in very late S-phase and immediately after micro-irradiation, antagonizes H3K20me3-53BP1-dependent DNA repair machinery (Fig. 10D). The PCNA protein seems to be the most important factor in this breakpoint and initiates HRR, because PCNA recruits to DNA lesions immediately after DNA injury, while BRCA1 appearance at the DSB sites is postponed to 20 min after local micro-irradiation, as we showed recently in [35].

**Figure 11:**
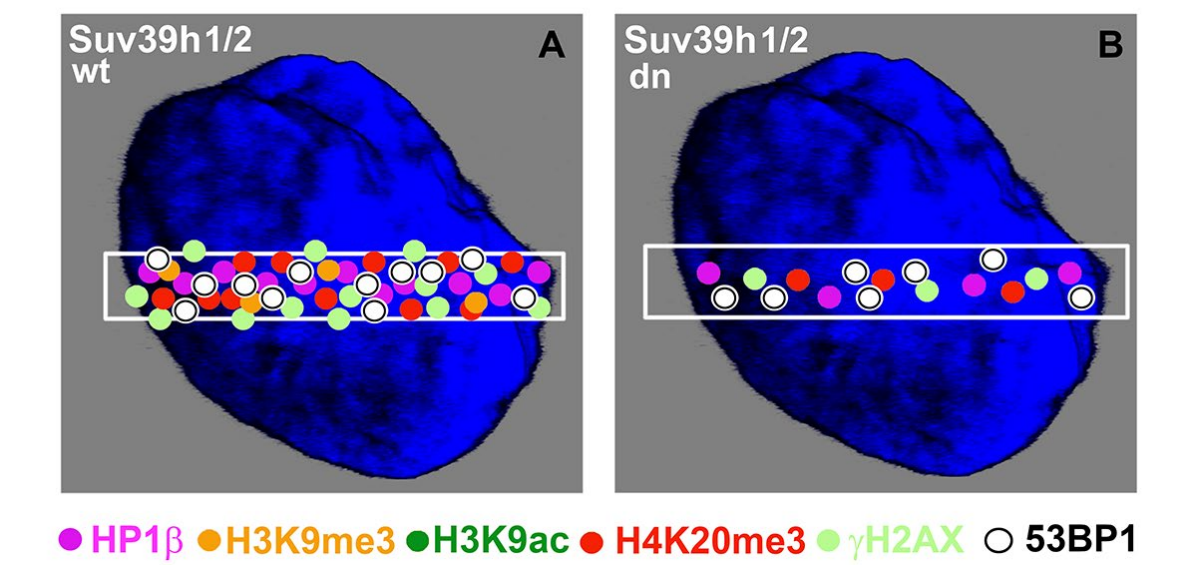
**Schematic illustration of protein levels in DNA lesions in wild-type and Suv39h1/h2 dn mouse embryonic fibroblasts.** Colored dots illustrate the levels of selected proteins at UVA-irradiated chromatin. The illustration demonstrates the appearance of the following proteins at DNA lesions: HP1β (pink circles), H3K9me3 (orange circles), H3K9ac (dark green circles), H4K20me3 (red circles), γH2AX (pale green circles), and 53BP1 (white circles). The selected micro-irradiated ROI is shown by a white rectangle. The figure represents an illustration of our results.

We additionally revealed that depletion of Suv39h1/h2 HMTs substantially reduces the level of not only H4K20me3 and 53BP1 but also HP1β protein at micro-irradiated chromatin (Fig. 3 Aa, b; 4C; 7 Aa, b; and summarized in Fig. 11A, B). From this view, Ayrapetov et al. [25] showed that a protein complex containing H3K9-HMT Suv39h1, HP1 and Kap-1 is rapidly recruited to DSB sites. Suv39h1 methylates H3K9, which is associated with activation of the Suv39h1/HP1/Kap-1 protein complex at damaged chromatin. This DDR-related nuclear event is mediated via the chromodomain (CD) of HP1 protein that recognizes H3K9me3. On the other hand, Luijsterburg et al. [31] claimed that all HP1 isoforms are recruited to UV-induced DNA lesions, and this DNA damage response is dependent on the chromo shadow domain (CSD) of the HP1 protein. Thus, this DDR-related nuclear event is independent of H3K9 tri-methylation. Here, we showed by immunoprecipitation that H3K9me3 interacts with the 53BP1 protein similarly to H4K20me3, and this interaction was not abrogated by Suv39h1/h2 depletion (Fig. 9A, B). The interactions between H3K9me3 and 53BP1 or H4K20me3 and 53BP1 imply a potential mechanism by which 53BP1 protein could form dimers. 53BP1 molecules (53BP1 dimerization or homo-oligomerization) might make bridges between H3K9me3 and H4K20me3, which is important for NHEJ repair mechanisms (Fig. 10C). The phenomenon that 53BP1 also recognizes chromatin with DSBs in the G2 phase [35] should be explained by the following: 53BP1 binds to not only H4K20 methylation but also to H3K9me3 (Fig. 9A, B) and H3K79 methylation, especially when the H4K20me2 level is reduced [36]. Especially H3K79me2 was revealed as the main histone target recognized by the 53BP1 protein at DSB sites [11,36,37]. Moreover, micro-irradiated chromatin in the G2 phase is not absent of H4K20me2/me3; thus, the level of H4K20me2 shown in Fig. 6 Ab and the level of H4K20me3 shown in Fig. 8C, D should be sufficient for the binding of 53BP1 to chromatin with DNA lesions that appear in the G2 phase of the cell cycle.

In contrast to H3K9me3, which is recognized by the 53BP1 protein, a key player in the NHEJ repair signaling, the HP1β protein did not interact with 53BP1 (Fig. 9A). Thus, HP1β is not a part of NHEJ-mediated DNA repair. In our previous work, we documented that HP1β recognizes UVA-damaged chromatin, which is positive on CPDs. Therefore, it seems likely that HP1β protein is functional in the NER pathway [32]. These results show that the function of HP1β and H3K9me3 in DDR is independent and is completely different from that of HP1β and H3K9 methylation when stabilizing heterochromatin or regulating gene silencing [38]. Our data also demonstrate the existence of a mixture of distinct DNA lesions induced by micro-irradiation: in our experimental system, when the cells are micro-irradiated by a UVA laser, we found both DSBs (recognized by the 53BP1-H3K9me3-H4K20me3 signaling pathway) and CPDs (characterized by recruitment of the HP1β protein that is involved in NER) [32].

Here, we also studied DNA repair processes from the view of histone acetylation (Fig. 3B). Hsiao and Mizzen [34] reported that H4K16 acetylation regulates the formation and dynamics of 53BP1 foci and that H4K16 antagonizes 53BP1 binding to H4K20me2 in transiently appearing double-strand breaks. Moreover, H4 deacetylation, which is responsible for 53BP1 focus formation and is associated with NHEJ, caused global gene silencing. These results fit well with our observation of a significant H3K9 deacetylation in UVA-damaged chromatin, which was not affected by Suv39h1/h2 depletion (Fig. 4 Ca, b, D). Polo and Jackson [33] summarized that DDR is also controlled by the histone acetylation status near DSB sites and that, for example, MOF-dependent acetylation of H4K16 supports the formation of IRIF, which is positive for MDC1, 53BP1 and BRCA1 proteins [39,40]. Thus, it seems likely that the efficiency of DNA repair is highly dependent on a complex epigenetic landscape, which consists of a well-known phosphorylation of histone H2AX, the specific acetylation status of N-terminal histone tails and, furthermore, H3K9me3 and H4K20 methylation (Fig. 10A-D). Moreover, we must not forget that epigenetic processes are enzymatically regulated; thus, related enzymes such as kinases, HATs, HDACs, HMTs, and HDMTs, also represent very fundamental regulatory units of the DNA damage response. For example, Southall et al. [41] proposed that enzymes other than Suv4-20h or SETD8 must catalyze H4K20 tri-methylation, and indeed, here, we showed that Suv39h1/h2 HMTs may also affect the level of H4K20 tri-methylation not only in DSB sites but also in chromocenters (Fig. 7 Aa, b; Ca-d; 11A, B).

## CONCLUSIONS

Altogether, we showed that UVA-damaged chromatin is simultaneously tri-methylated on histones H3K9 and H4K20 and that this DDR-related epigenetic process is Suv39h1/h2 dependent. Our results additionally suggest a co-regulatory function among H3K9me3, H4K20me3 and Suv39h1/h2 HMTs at DNA lesions that are recognized by the 53BP1-mediated NHEJ repair mechanism. This DNA repair nuclear event is independent of the function of the HP1β protein and preferentially appears in the G1 phase of the cells cycle. However, when the cells enter very late S phase, DNA repair mediated via the H3K9me3-H3K20me3-53BP1 protein complex is replaced by PCNA-BRCA1 repair machinery.

## MATERIALS AND METHODS

### Cell cultivation and treatment

Wild-type (wt) and Suv 39h1/h2 double-knockout (dn) mouse embryonic fibroblasts (MEFs; a gift from Prof. Thomas Jenuwein, Max Planck Institute of Immunology and Epigenetics, Freiburg, Germany) were cultivated in Dulbecco’s Modified Eagle’s Medium supplemented with 10% fetal calf serum (FCS) (Merck, Darmstadt, Germany). The medium for Suv39h1/h2 dn also included 1 μl β-mercaptoethanol (#31350-010, Thermo Fisher Scientific, Waltham, Massachusetts, USA), 5 ml nonessential amino acids (100 ×; #1140-035, Thermo Fisher Scientific), 5 ml sodium pyruvate (#11360-039, ThermoFisher Scientific, Waltham, Massachusetts, USA), and 1.5 g NaHCO_3_. The cells were immortalized by repeated *in vitro* cultivation [27]. HAP1 cells were purchased from Horizon Discovery Company. This near-haploid cell line was derived from the KBM-7 cell line. The cells are characterized by a 244-bp insertion in exon 2 of the gene for human SUV39h1 HMT, and these cells were grown in Iscove's Modified Dulbecco's Medium (IMDM) (#12440053, ThermoFisher Scientific, USA) supplemented with 10% FCS, 100 U/ml penicillin and 100 μg/ml streptomycin. HeLa Fucci cells were cultivated following Suchánková et al. [35].

### Cell transfection with plasmid DNA

For live-cell studies, we used the following plasmids: GFP-tagged HP1β [42]; mCherry-tagged 53BP1 (mCherry-BP1-2 pLPC-Puro (a fragment of human 53BP1, aa 1220 - 1711; #19835, Addgene, Cambridge, Massachusetts, USA), mCherry-tagged PCNA (a generous gift from prof. Christina Cardoso, Technical University, Darmstadt, Germany), pDEST-FRT/T0-GFP-BRCA1 (#71116, Addgene, USA), and GFP-tagged JMJD2b (termed GFP-JMJD2b-1086), (a generous gift from prof. Thomas Jenuwein and Dr. Nicholas Shukeir, Max Planck Institute of Immunobiology, Freiburg, Germany). The plasmids were introduced into *E. coli* DH5α, and the DNA was isolated using the Qiagen Plasmid Maxi Kit (#121693; QIAGEN, Bio-Consult, Praha, Czech Republic). The cells were transfected with 2-5 μg plasmid DNA using METAFECTANE (#T020–1.0, Biontex Laboratories GmbH, München, Germany) [35].

### Immunofluorescence staining

The cells were fixed in 4% paraformaldehyde (PFA) for 10 min at room temperature (RT), permeabilized with 0.2% Triton X-100 (Merck) for 10 min and 0.1% saponin (Merck) for 12 min, and washed twice in phosphate-buffered saline (PBS) for 10 min. Bovine serum albumin (Merck) (1% dissolved in PBS) was used as a blocking solution. Slides with fixed cells were washed for 15 min in PBS and were incubated with the following antibodies: anti-phosphorylated histone H2AX (γH2AX; phospho S139, #ab2893, Camridge, Abcam), γH2AX (phospho S139, #ab 22551, Abcam), anti-53BP1 (#ab21083, Abcam), anti-histone H3K9ac (#06-942, Merck), anti-histone H3K9me3 (#ab8898, Abcam), H4K20me1 (A2370 Abclonal, Woburn, MA, USA), H4K20me2 (A-4047-025 Epigentek, Lab Mark a.s., Prague Czech Republic), and the anti-histone H3 (phospho S10) antibody (#ab5176, Abcam). This procedure was modified for the antibody against histone H4K20me3 (#A-4048-050, Epigentek, Lab Mark a.s.). The cells were also fixed with 2 ml of 4% PFA for 10 min, followed by the addition of 100 ml of 1% SDS after 5 min of fixation. Additionally, the Triton X concentration was increased to 0.3%, while saponin was not used in this protocol. The primary antibodies were diluted 1:200 in 1% bovine serum albumin (BSA) in PBS. After overnight incubation, the appropriate secondary antibodies were applied. We optimized the use of the following secondary antibodies: Alexa 488-conjugated goat anti-rabbit (#ab150077, Abcam), Alexa 594-conjugated goat anti-rabbit (#A11037, ThermoFisher Scientific), Alexa 488-conjugated goat anti-mouse (#A11029, ThermoFisher Scientific), Alexa 647-conjugate goat anti-rabbit (#A21245, ThermoFisher Scientific) and Alexa 405-conjugated goat anti-mouse (#A31553, ThermoFisher Scientific). The samples were incubated without primary antibodies for negative control staining. The secondary antibodies were diluted at 1:200 in PBS containing 1% BSA. The DNA content was visualized using 4′,6-diamidino-2-phenylindole (DAPI; Merck, Germany), and Vectashield (Vector Laboratories, Burlingame, CA, USA) was used as the mounting medium.

### Immunoprecipitation

To investigate 53BP1-H3K9me3, 53BP1-H4K20me2, 53BP1-H4K20me3 and 53BP1-HP1β interactions, Suv39h1 (wt) and Suv39h1 (dn) cells were grown to 70% confluence, and then, entire cell populations were irradiated with 5 Gy of γ-rays delivered by cobalt-60 (Chirana, Czech Republic). Then, 24 hours after γ-irradiation, cells were washed in PBS buffer and incubated in Pierce^TM^ IP Lysis Buffer (# 87788, Thermo Fisher Scientific Inc.), supplemented with a protease inhibitor cocktail [1 mM phenylmethylsulfonyl fluoride (PMSF) and 1 μg/ml aprotinin] for 5 min on ice. The total protein concentration was determined by DC protein assay kit (#5000111, Bio-Rad, Bio-Consult, Prague, Czech Republic) and by ELISA Reader μQuant (BioTek, Winooski, VT, USA). Immunoprecipitation was performed according to the manufacturer's protocol (Catch and Release®v2.0 Reversible Immuno-precipitation System, #17-500, Merck). Briefly, spin columns with resin were washed twice with 1x Wash Buffer; after that, the reagents were added to the spin Columns in the following order: 1x Wash Buffer, ceell lysate, specific primary antibody against 53BP1 (#ab21083, Abcam) or negative control antibody (IgG whole molecule, #A4914 Merck), and Antibody Capture Affinity Ligand. Immunoprecipitation reactions were performed overnight at 4 °C. Next-day Spin Columns were washed three-times with 1x Wash Buffer and proteins were eluted from columns by 1x Denaturing Elution Buffer containing β-mercapto-ethanol (to a final concentration of 5%). Precipitates were fractionated by SDS-PAGE of western blot.

### Western blotting

Western blotting was performed using the methods reported by Legartová et al. (2014) and Franek et al. (2016) [43,6]. We used the following primary antibodies: anti-53BP1 (#ab21083, Abcam), anti-α-tubulin (#ab80779, Abcam), anti-MDC1 (#ab11169, Abcam), anti-MDC1 (#ab41951, Abcam), anti-phosphorylated histone H2AX (γH2AX; phospho S139; #ab2893, Abcam), anti-HP1β (#MAB3448, Merck), anti-H3 (#ab7091, Abcam), anti-H3K9me1 (#ab9045, Abcam), anti-H3K9me2 (#ab1220, Abcam), anti-H3K9me3 (#ab8898, Abcam), anti-histone H3K9ac (#06-942, Merck), anti-histone H4K20ac (#701778, ThermoFisher Scientific), anti-histone H4K20me2 (#A-4047-050, Epigentek, Lab Mark a.s.), anti-histone H4K20me3 (#A-4048-050, Epigentek, Lab Mark a.s.). As secondary antibodies, we used anti-rabbit IgG (#A-4914, Merck, Germany; dilution 1:2000), anti-mouse IgG (#A-9044, Merck; dilution 1:2000) and anti-mouse IgG1 (#sc-2060, Santa Cruz Biotechnology, Dallas, Texas, USA; dilution 1:1000).

### Flow cytometric analysis of the cell cycle

For cell cycle analysis, the confluence of Suv39h1/h2 wt and Suv39h1/h2 dn cells, exposed to γ-irradiation, was 60-80%. Twenty-four hours after γ-irradiation (5 Gy), the cells were harvested using trypsin, followed by washing the cells in cold phosphate-buffered saline (PBS). Next, the cells were re-suspended in 0.5 ml of PBS at 4°C and fixed in 4 ml of 70% ethanol (at 4°C) for at least 30 minutes. Cell nuclei were stained with Vindelov’s solution consisting of 1 mM Tris-Cl (pH 8.0), 1 mM NaCl, 0.1% Triton X100, 10 mg/ml RNase A and 5 µg/ml propidium iodide (PI) [5]. Staining was performed at 37°C for 30 min, and then, the cells were washed 3× in PBS. Cell cycle profiles were measured using a BD FACSVerse flow cytometer (BD Biosciences, Franklin Lakes, NJ, USA) equipped with a 488-nm laser. Emission of PI was detected using a detector with a 586/42 filter. For cell cycle quantification, we used FACSuite software (BD Biosciences, Franklin Lakes, NJ, USA) and ModFit software (Verity Software House, Topsham, USA). For measurement, we prepared three independent biological replicates that were analyzed three times.

### Laser scanning confocal microscopy and image analysis

Laser scanning confocal microscopy was performed using a Leica TCS SP5-X microscopic system (Leica, Mannheim, Germany). We used a white light laser (WLL; wavelengths of 470-670 nm in 1-nm increments) to acquire fluorescence images. Cross-talk between the fluorochromes was eliminated by application of the sequential scanning mode of the LEICA LAS AF software. For observation and image acquisition, we used a 63× oil objective (HCX PL APO, lambda blue) with a numerical aperture (NA) = 1.4. A detailed description of the image acquisition is provided in [32,35,44].

### Induction of DNA lesions by local micro-irradiation

For the micro-irradiation experiments using UVA lasers (355-nm), cells were seeded on 35-mm grid-500 μ-dishes (#81166, Ibidi, Madison, Wisconsin, USA) at 70% confluence and were pre-sensitized with 10 μM BrdU for 16 to 18 h. Cells were cultured in an incubation chamber (EMBL) at 37°C with 5% CO_2_. In the selected cell nuclei, we irradiated only the defined region of interest (ROI). Micro-irradiation by UVA laser, connected to a Leica SP5 X confocal microscope (Leica Microsystems), was performed at 15 mW. LEICA LAS AF software was used for image acquisition and analysis. For the optimization of experiments and verification of protein recruitment to DNA lesions, we analyzed GFP-tagged HP1β [45]. Next, we studied the accumulation of mCherry-tagged 53BP1 to DNA lesions, and H3K9ac, H4K20me1, H4K20me2, and H4K20me3 at locally micro-irradiated chromatin in living and fixed Suv39h1/h2 wt and Suv39h1/h2 dn cells. After the immunostaining procedure, locally micro-irradiated cells were found on gridded microscope dishes according to registered coordinates. Image acquisition was performed at a resolution of 1024×1024 pixels before micro-irradiation. The microscope settings for DNA damage experiments were as follows: 512×512-pixel resolution, 400 Hz, bidirectional mode, 64 lines, zoom > 8×.

### Irradiation by γ-rays

Cells were also irradiated with 5 Gy of *γ-*rays delivered by cobalt-60 (source Chisostat, Chirana, Czech Republic). The cells were harvested 30 minutes and 24 hours after irradiation and were fixed for further analysis by western blotting, immunofluorescence, and confocal microscopy.

### Statistical analyses and quantification of fluorescence intensity

The densities of the western blot fragments and immunofluorescence signals were quantified by ImageJ and ImageQuant TL (Typhoon 9000 software). The collected data were then normalized (relative to standard densities of histone H3 or α-tubulin). The results of the statistical analysis were further processed in Excel software, and data are shown in Excel graphs. We used the online tool http://www.socscistatistics.com/tests/studentttest/Default2.aspx or Sigma Plot software for Student’s t-test and http://shiny.chemgrid.org/boxplot/ for data plotting. We analyzed the intensity of fluorescence across selected regions of interest (ROIs) in the cell nuclei and foci number calculation using Leica software (LAS X; 3D-analysis mode). To analyze the number of foci, 50-60 cell nuclei were chosen, and the intensity of fluorescence in ROI was measured for 25-30 cell nuclei. The relative fluorescence intensity refers to the intensity of fluorescence in micro-irradiated ROI, normalized to the fluorescence intensity outside micro-irradiated ROI.
